# Severe bronchiolitis before and after the COVID-19 pandemic: a retrospective database analysis by the Italian Network of PICU study group (TIPNet)

**DOI:** 10.1186/s44158-024-00210-1

**Published:** 2024-11-26

**Authors:** Francesca Izzo, Rosanna I. Comoretto, Angela Amigoni, Marco Daverio, Elena Zoia, Veronica Diotto, Francesco Sacco, Claudio Nettuno, Anna Tessari, Enzo Picconi, Maria Cristina Mondardini, Gaia Milvia Bregant, Andrea Wolfler, Dario Gregori, Anna Camporesi

**Affiliations:** 1 Anesthesia and Intensive Care Unit, Buzzi Children’s Hospital, Milan, Italy; 2 Department of Public Health and Pediatrics, University of Turin, Turin, Italy; 3 Pediatric Intensive Care Unit, Department of Woman’s and Child’s Health, University Hospital, Padua, Italy; 4 Department of Neonatal and Pediatric Intensive Care, University Hospital, Verona, Italy; 5 Pediatric Intensive Care Unit, Children’s Hospital C. Arrigo, Alessandria, Italy; 6 Pediatric Intensive Care Unit, Fondazione Policlinico Universitario “A. Gemelli” IRCCS, Rome, Italy; 7 Pediatric Intensive Care Unit, IRCCS Azienda Ospedaliero Universitaria Di Bologna, Bologna, Italy; 8 Anesthesia and Intensive Care Unit, Institute for Maternal and Child Health, IRCCS “Burlo Garofolo”, Trieste, Italy; 9 Department of High Intensity Care and Surgery, IRCCS Giannina Gaslini Institute, Genoa, Italy; 10 Unit of Biostatistics, Epidemiology and Public Health, Department of Cardiac, Thoracic, Vascular Sciences and Public Health, Padua, Italy

**Keywords:** Bronchiolitis, Non-invasive ventilation, Intubation, Pediatric intensive care unit, Respiratory syncytial virus (RSV), Severity

## Abstract

**Background:**

The first post-COVID-19 pandemic year demonstrated an unusual bronchiolitis epidemic in both hemispheres and has been attributed to the removal of barriers implemented during SARS-CoV-2 infection. Several countries reported an increase in respiratory syncytial virus (RSV) bronchiolitis, with more hospitalizations and a greater need for respiratory support. We aimed to evaluate the consequences of the COVID-19 pandemic on the epidemiology and management of severe bronchiolitis in pediatric intensive care units (PICUs) in Italy.

**Methods:**

Multicenter, retrospective, cohort database analysis. All children younger than 24 months admitted to 7 PICUs from October 2017 to April 2023 diagnosed with bronchiolitis were included. We compared patients from pre-COVID and post-COVID eras, excluding patients from the 2020–2021 season due to low numbers. Logistic regression models were used to assess the impact of the pre-/post-COVID period on the need for invasive ventilation.

**Results:**

Seven hundred fifteen patients were admitted to PICU during the study period, 451 patients pre-COVID and 251 patients post-COVID. Patients in the post-COVID group were older, had more comorbidities, and had higher Pediatric Index of Mortality scores at admission but the need for respiratory support was not significantly different. There was high variability in bronchiolitis management across centers. Presenting pre-COVID was protective against the risk of mechanical ventilation, adjusted for age and disease severity at admission (OR 0.38, 95% CI 0.16–0.89), while RSV infection increased the risk of intubation (OR 2.49, 95% CI 1.1–5.63).

**Conclusions:**

PICUs have faced an unexpected peak of significantly more severe cases of bronchiolitis after the COVID-19 pandemic, which did not require increased respiratory support.

**Supplementary Information:**

The online version contains supplementary material available at 10.1186/s44158-024-00210-1.

## Introduction

Bronchiolitis is a common viral lower respiratory tract infection characterized by acute small airway inflammation and represents the leading cause of hospital admission in infants and young children [1, 2]. In temperate regions, bronchiolitis shows a seasonal pattern, characterized by an increase in the number of cases in late October, a peak in January/February, and an end in April [3]. The most severe cases are usually secondary to respiratory syncytial virus (RSV), which infects a large proportion of children less than 2 years of age [4, 5]. Severe bronchiolitis is the main cause of admission to the pediatric intensive care unit (PICU) and is characterized by airway obstruction, hypoxemia, increased work of breathing, and respiratory distress, which may require advanced supportive management, including hydration, oxygen, or mechanical ventilation [6–8]. During the first year of the COVID-19 pandemic (2020), several authors reported a significant reduction in hospital admissions for bronchiolitis compared to previous years, likely due to the extraordinary reduction in interpersonal contacts resulting from SARS-CoV-2 safety measures [9–11]. During the following year, countries gradually reduced the distancing rules and restored a normal social and working life, aided by the initial Coronavirus vaccination campaign. The exponential increase in human contact led to a subsequent rise in common viral infections, including those caused by RSV [12]. This phenomenon was first described in Australia in 2020 when an unseasonal bronchiolitis epidemic was observed just after the relaxation of social distancing measures [13]. In Italy, an unexpected increase in hospitalized bronchiolitis was observed since the end of 2021 in many centers, but the need for respiratory support was not clear [14–16].

Our study aimed to compare the epidemiology, disease course, and management of severe bronchiolitis before and after the COVID-19 pandemic in seven Italian PICUs and to identify possible risk factors for mechanical ventilation.

## Methods

### Study design and setting

This is a multicenter, retrospective, database analysis based on a prospectively compiled electronic web-based national registry of the Italian Network of PICU Study Group (TIPNet).

TIPNet is a national research network established in 2010, which includes 17 Italian PICUs. The registry’s use for nonprofit research purposes has been approved by the Ethics Committee of the Buzzi Hospital, the coordinating registry center (approval number 269–052014 of 23/05/2014 and number 0047897 of 16/11/2020). Data were collected and managed using the REDCap (Research Electronic Data Capture) platform hosted at the Unit of Biostatistics, Epidemiology, and Public Health of the University of Padua [17, 18]. The guidelines for reporting observational studies according to the Strengthening the Reporting of Observational Studies in Epidemiology (STROBE) Statement were followed [19].

### Inclusion criteria and outcome

We considered the completeness of the data entered relative to the admissions conducted by each center as an inclusion criterion for the study and we established a cut-off of 90% complete data for each patient. Seven out of 17 PICUs met this criterion.

All patients younger than 24 months of age admitted to these PICUs from 2017 to 2023 with a diagnosis of bronchiolitis were included. The period spanned from October 1st to April 30th, which is the typical bronchiolitis season in Italy.

The cohort was subsequently divided into a pre-COVID period (patients admitted in 2017–2018, 2018–2019, and 2019–2020 seasons) and a post-COVID period (those admitted during the 2021–2022, and 2022–2023 seasons). The 2020–2021 season was intentionally excluded due to the low number of patients admitted, to reduce confounding and potential biases. Therefore, it was intended as a useful time point to separate the pre- and post-COVID periods.

The primary outcome was defined as the proportion of infants requiring invasive mechanical ventilation (IMV) in the pre-COVID and post-COVID groups. The secondary outcomes were the assessment of disease severity at admission, type, and duration of invasive and non-invasive ventilation (NIV), and PICU length of stay (LOS) in the two study periods. NIV was defined as positive airway pressure delivered through a nasal mask, nasal prongs, a facial mask, or a helmet. NIV ventilation modes were classified as follows: continuous positive airway pressure (CPAP), pressure support ventilation (PSV), bilevel positive airway pressure (BiPAP), and assisted pressure control ventilation (A-PCV). Mechanical ventilation was defined as invasive mechanical ventilation (IMV). High flow nasal cannula (HFNC) was defined as 2 L·kg^−1^·min^−1^ flow of a gas oxygen mixture through nasal cannula and coded separately from non-invasive support. Each PICU was classified as “small PICU” if it had fewer than six beds and as a “large PICU” if it had six or more beds.

### Data collection

The following data were collected for each patient: age, gender, weight (kg), comorbidities, history of prematurity (< 36 weeks of gestational age), admission to a small/large PICU, Pediatric Index of Mortality-3 (PIM-3) score [20] at admission, inspired oxygen fraction and lactates at admission, use of HFNC, use of respiratory support (IMV only, NIV only or both) and its duration (in days), type of NIV interface used, use of high-frequency oscillatory ventilation (HFOV) and surfactant or neuromuscular blocking agents (NMB), type of microorganism isolated, presence of co-infection (two microorganisms isolated) or bacterial infection, PICU LOS in days, and in-hospital mortality.

### Statistical analysis

Descriptive data are presented as absolute numbers and percentages for categorical variables and as median and interquartile ranges (IQRs) for continuous ones. Chi-square and Kruskal–Wallis tests, as appropriate, were used to evaluate significant differences in sample characteristics in the two periods considered (pre-COVID and post-COVID). Univariable logistic regression models were used to assess the association between IMV and the main patient characteristics, including the admission period. Multilevel logistic regression models were then used to assess the potential association between IMV and the admission period, considering the hierarchical structure of the data and including a random effect to account for the variability between centers. Results were reported as odds ratios (ORs) and 95% confidence intervals (CIs). Models’ goodness-of-fit was assessed with the Nagelkerke *R*^2^ [21] and the model performance was evaluated using the area under the receiver operating characteristics curve (AUROC). All statistical tests were two-sided and the level of statistical significance was set at 0.05. All analyses were conducted using R software (version 4.2.2) [22].

## Results

**Fig. 1:**
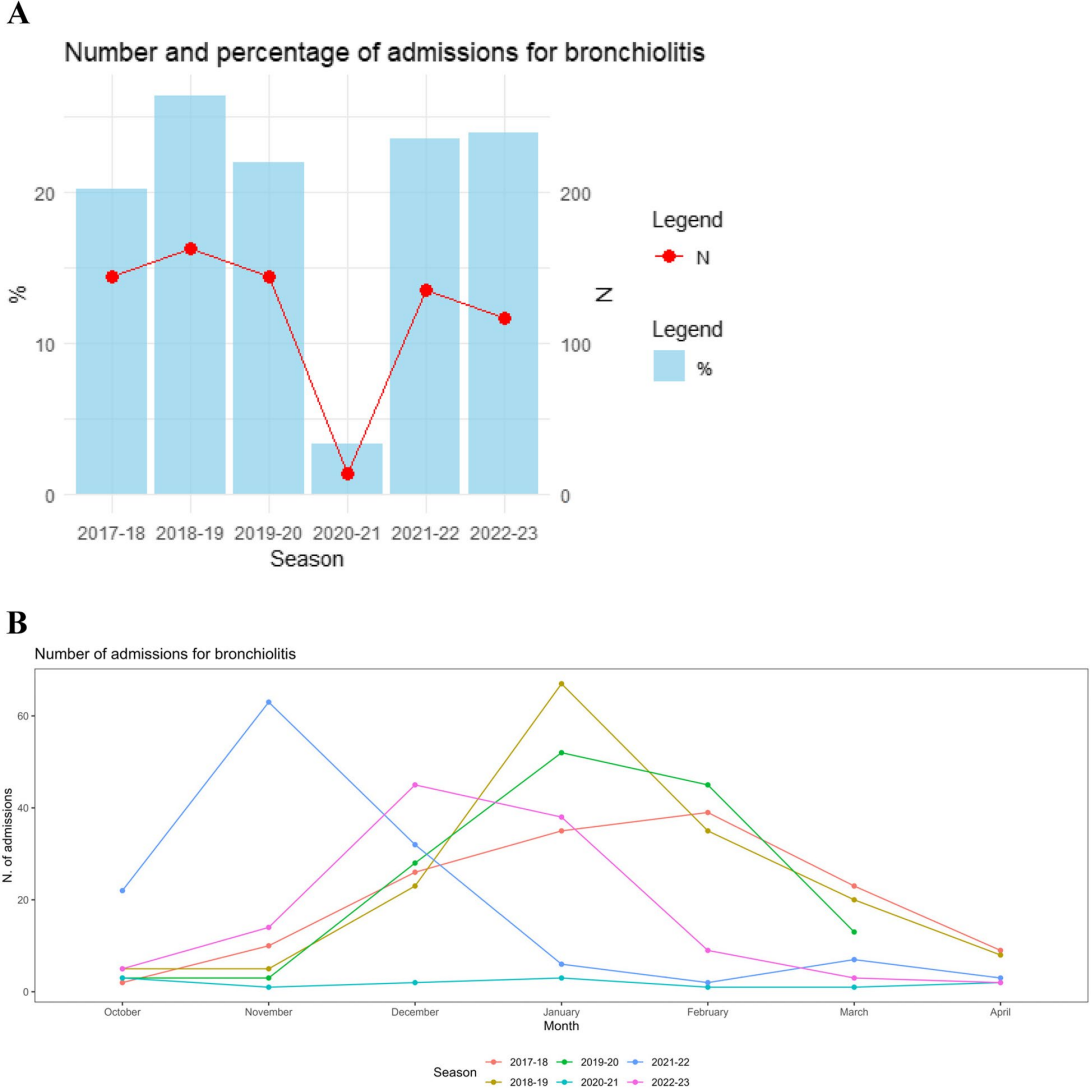
Number of admissions by season (**A**) and by month (**B**)

In total, 715 patients with a diagnosis of bronchiolitis were admitted to the seven PICUs during the six seasons considered. The seasonal admission numbers and percentages were relatively consistent across all seasons except for the 2020–2021 COVID-19 season, during which there was a tenfold reduction (Fig. 1A). Excluding 13 patients from the 2020–2021 season, the remaining 702 patients were grouped according to the period of admission, with 451 patients admitted to PICU in the pre-COVID period and 251 in the post-COVID period. The temporal distribution of admissions is depicted in Fig. 1 (Fig. 1B).

### Epidemiology and severity at admission

In the post-COVID period, children admitted to the PICU were generally older (73 days [IQR 40–178] vs 55 days [IQR 33–125], *p* = 0.039) and presented with a significantly worse PIM-3 score at admission (median 0.52% [IQR 0.38–0.67] vs 0.46% [IQR 0.37–0.62], *p* = 0.046). They also had more comorbidities (21% vs 13%, *p* = 0.039): chronic respiratory conditions (4.4%), congenital cardiac disease (3.7%), neurologic (3.1%), and syndromic disorders (3.1%) were the most common underlying conditions in both periods (Table 1). RSV cases made up 82% of the sample and did not change over time. Bacterial infection and coinfection with two microorganisms occurred in 7% and 15% of the overall sample, respectively. The PICU LOS was not significantly different in the two periods considered (median 4.0 days [IQR 3.0–7.0] vs 5 days [IQR 3.0–8.0], *p* = 0.373). Sepsis occurred in 1% of cases and overall mortality was 0.3%.

**Table 1:** Characteristics of the sample by period

Characteristic	*N*	Pre COVID, *N* = 451	Post COVID, *N* = 251	Overall, *N* = 702	*p*-value
Large center		246 (55%)	127 (51%)	373 (53%)	0.300
Gender (M)	702	234 (52%)	147 (59%)	381 (54%)	0.144
Age (d), median (IQR)	702	55 (33, 125)	73 (40, 178)	62 (34, 142)	**0.039**
Ethnicity	702				0.236
Caucasian		343 (76%)	180 (72%)	523 (75%)	
Arabic		55 (12%)	29 (12%)	84 (12%)	
African		20 (4.4%)	17 (6.8%)	37 (5.3%)	
Asiatic		22 (4.9%)	12 (4.8%)	34 (4.8%)	
Hispanic		8 (1.8%)	6 (2.4%)	14 (2.0%)	
Mixed ethnicity		3 (0.7%)	6 (2.4%)	9 (1.3%)	
Afro-American		0 (0%)	1 (0.4%)	1 (0.1%)	
Weight (kg), median(IQR)	700	4.60 (3.70, 6.30)	5.00 (4.00, 6.95)	4.80 (3.80, 6.50)	0.056
Comorbidities	702	59 (13%)	52 (21%)	111 (16%)	**0.039**
Ex-premature	702	61 (14%)	20 (8.0%)	81 (12%)	0.056
Respiratory	702	18 (4%)	13 (5.2%)	31 (4.4%)	0.5
Cardiac	702	17 (3.8%)	9 (3.6%)	26 (3.7%)	> 0.9
Neurologic	702	17 (3.8%)	5 (2.0%)	22 (3.1%)	0.2
Syndromic	702	13 (2.9%)	9 (3.6%)	22 (3.1%)	0.6
PIM 3 score (%), median (IQR)	673	0.46 (0.37, 0.62)	0.52 (0.38, 0.67)	0.48 (0.37, 0.63)	**0.046**
FiO_2_ at admission, median(IQR)	394	0.35 (0.30, 0.40)	0.40 (0.30, 0.45)	0.40 (0.30, 0.40)	0.052
Lactates at admission (mmol/L) median(IQR)	297	1.40 (1.00, 2.12)	1.38 (0.90, 2.20)	1.40 (1.00, 2.20)	0.325
RSV cases	538	287 (84%)	155 (79%)	442 (82%)	0.465
Sepsis	538	1 (0.2%)	3 (1%)	4 (1%)	0.187
Bacterial infection	538	16 (5%)	21 (11%)	37 (7%)	0.800
Co-infection (2 microorganisms isolated)	538	33 (10%)	48 (25%)	81 (15%)	0.600

*Abbreviations*: *PIM* Pediatric Index of Mortality, *FiO2* fraction of inspired oxygen, *RSV* respiratory syncytial virus. Column *N* reports the number of records available for each variable. Boldface: significant p-values

### Respiratory support and adjunctive therapies

Data on respiratory support are reported in Table 2. NIV was used in 70% of the patients and the overall rate of IMV (alone or before and/or after NIV) was 17%.

**Table 2:** Comparison between respiratory support and adjunctive therapies in pre-COVID and post-COVID groups

Characteristic	*N*	Pre-COVID, *N* = 451	Post-COVID, *N* = 251	Overall, *N* = 702	*p*-value
HFNC	702	222 (49%)	134 (53%)	356 (51%)	0.360
NIV	702	320 (71%)	174 (69%)	494 (70%)	0.672
IMV	702	78 (17%)	38 (15%)	116 (17%)	0.626
NIV ventilation modes:					
PSV/BiPAP	469	73 (26%)	101 (55%)	174 (37%)	** < 0.001**
CPAP	469	126 (44%)	56 (31%)	182 (39%)	0.012
A-PCV	469	85 (30%)	26 (14%)	111 (24%)	** < 0.001**
NIV interfaces:					
Nasal mask	702	70 (16%)	86 (34%)	156 (22%)	** < 0.001**
Facial mask	702	18 (4.0%)	3 (1.2%)	21 (3.0%)	0.086
Nasal prongs	702	224 (50%)	84 (33%)	308 (44%)	** < 0.001**
Helmet	702	47 (10%)	33 (13%)	80 (11%)	0.360
Full face	702	33 (7.3%)	7 (2.8%)	40 (5.7%)	0.031
NIV complications	297	1 (0.9%)	1 (0.5%)	2 (0.7%)	0.999
NIV failure	534	31 (8.8%)	11 (6%)	42 (7.9%)	0.400
Chest tube positioning	702	4 (0.9%)	2 (0.8%)	6 (0.9%)	0.999
HFOV	116	8 (10%)	1 (2.6%)	9 (7.8%)	0.360
NMB	115	24 (31%)	13 (35%)	37 (32%)	0.672
Surfactant	117	10 (13%)	2 (5.1%)	12 (10%)	0.400
Extubation failure	116	4 (3%)	2 (2%)	6 (5%)	0.999
NIV length (d), median(IQR)	567	3.00 (2.00, 5.00)	3.00 (2.00, 4.00)	3.00 (2.00, 5.00)	0.360
IMV length (d), median(IQR)	115	11 (7, 14)	8 (5, 13)	10 (6, 14)	0.125
Antibiotic therapy	648	246 (56%)	138 (65%)	384 (59%)	0.086
PICU LOS (d), median(IQR)	702	5.0 (3.0, 8.0)	4.0 (3.0, 7.0)	4.0 (3.0, 7.0)	0.373
Mortality	702	0 (0%)	2 (0.8%)	2 (0.3%)	0.260

*Abbreviations*: *HFNC* high flow nasal cannula, *NIV* non-invasive ventilation, *IMV* invasive mechanical ventilation, *PICU* pediatric intensive care unit, *PSV* pressure support ventilation, *BiPAP* bilevel positive airway pressure, *CPAP* continuous positive airway pressure, *A-PCV* assisted pressure controlled ventilation, *HFOV* high-frequency oscillatory ventilation, *NMB* neuromuscular blockade, *LOS* length of stay. Boldface: significant p-values

The use of HFNC and the need for NIV and its duration did not show significant changes between the two periods. Nasal prongs (44%) and nasal masks (22%) were the two interfaces more commonly used. During the pre-COVID period, nasal prongs were more frequently used (50% vs 33%, *p* < 0.001), whereas nasal masks were more commonly used during the post-COVID period (34% vs 16%, *p* < 0.001).

NIV modes also changed with time, with PSV or BiPAP being used more frequently in the Post-COVID era (55% vs 26%, *p* < 0.001), while CPAP and A-PCV were mainly used in the Pre-COVID period (*p* < 0.05 in both cases). The rate of NIV failure and complications remained consistently lower than 10% and 1%, respectively.

**Fig. 2:**
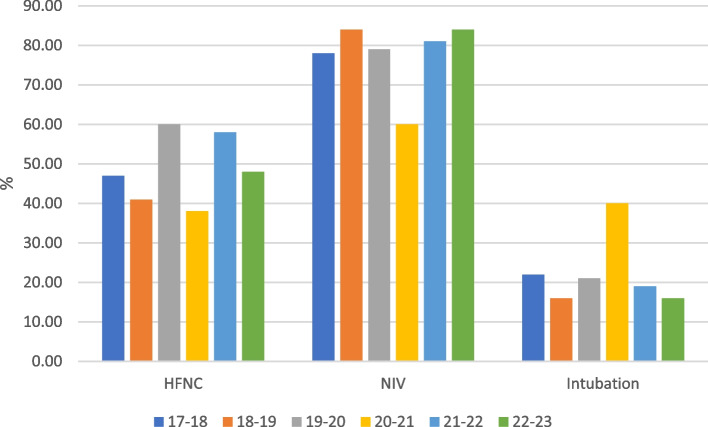
Seasonal differences in respiratory support. Abbreviations: HFNC, high flow nasal cannula; NIV, non-invasive ventilation

The rate of IMV use was comparable between the two periods (15% vs 17%) and the extubation failure was very low overall (5%). The seasonal comparison of respiratory support (Fig. 2) confirmed the same trends, with substantial stability in the need for NIV and IMV over time.

The use of HFOV, NMB, and surfactant in intubated patients did not change in the two periods considered, nor did the rate of chest tube placement, which was always very low (0.9%).

### Comparison between centers

The clinical characteristics of patients admitted for bronchiolitis according to the center are reported in Supplementary Table S1. There was huge heterogeneity across centers in terms of both case mix and management practices. Some PICUs admitted predominantly younger children (median 50 days, IQR 34–104) compared to others (median 125 days, IQR 35–347). The PIM-3 ranged from 0.16% (IQR 0.12–0.51) to 0.53% (IQR 0.45–0.68). Using the average risk profile for intubation, the probability of a child with bronchiolitis being intubated varied from 4.7 to 50% across units. Even NIV rates, modalities, and interfaces used varied widely.

### Risk factors for intubation

Univariable logistic regression models assessed the association between mechanical ventilation and patient characteristics (Supplementary Table S2). These results showed that patients’ age, ex-prematurity, and the use of facial masks were associated with a lower risk of intubation outcomes. Conversely, the use of nasal masks, nasal prongs, and helmets were risk factors for a subsequent IMV.

**Fig. 3:**
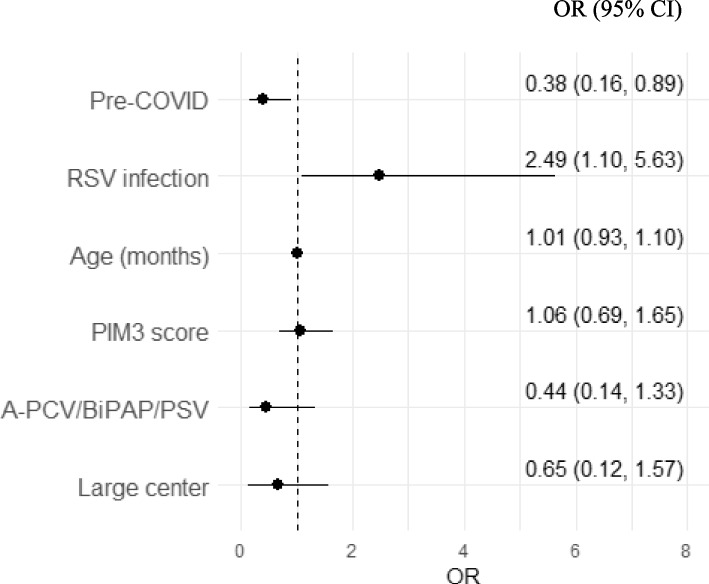
Multivariable logistic regression models for intubation outcome. Model fit indexes: ICC (intraclass correlation coefficient) = 0.22; Nagelkerke *R*^2^ = 0.645; AUC = 0.76 (95% CI 0.70–0.81). Abbreviations: OR, odds ratio; CI, confidence interval; PIM, Pediatric Index of Mortality; RSV, respiratory syncytial virus; PSV, pressure support ventilation; BiPAP, bilevel positive airway pressure; A-PCV, assisted pressure controlled ventilation

Considering the hierarchical structure of the data, the best multilevel regression model for intubation outcome (Fig. 3) included the study period, patient’s age and severity (PIM-3), PSV/BiPAP/A-PCV ventilation modes and the size of PICU (large centers vs small ones). In this model, the pre-COVID period represents a protective factor towards the risk of IMV (OR 0.38, 95% CI 0.16–0.89). Furthermore, RSV infection was associated with a higher risk of mechanical ventilation (OR 2.49, 95% CI 1.10–5.63). PIM3 score, age, the use of A-PCV/BiPAP/PSV modes, and the size of the center were not associated with the risk of intubation. For this model, the intraclass correlation coefficient (ICC) of 0.22 indicates that approximately 22% of the total variance is attributable to differences between centers, and the variables entered into the model explained 65% of the variance of the outcome (Nagelkerke *R*^2^ = 0.645). Moreover, the model showed good accuracy, with an AUC of 0.76 (95% IC 0.70–0.81).

## Discussion

Our study shows the possible impact of the COVID-19 pandemic on the epidemiology of severe bronchiolitis in Italian PICUs, revealing a shift towards older children and an association with a higher risk of mortality. Nonetheless, the need for NIV and IMV remained unchanged over the last 6 years, although the post-COVID period and RSV infection have been identified as risk factors for intubation.

The 2020–2021 winter season showed a tenfold reduction in PICU admissions due to bronchiolitis in Italy, consistent with findings from other countries around the world [9–11]. Traditionally, RSV is thought to be spread by older siblings, typically aged 1–5 years [23]. During the winter of 2020–2021 in Italy, as in other countries, this age group largely stayed at home due to social restrictions, further supporting their role in spreading the disease.

The following year saw a resurgence of PICU admissions for bronchiolitis in both hemispheres, with anticipated or even unusual rise [24–26] in non-usual months. It has been hypothesized that the lack of exposure to RSV during the first pandemic year may have led to an “immunity debt”, resulting in a more severe course of the disease [27] or affecting an older population [13, 28] that had previously lacked exposure. In our cohort, we observed a peak in admissions earlier than the typical Italian bronchiolitis season during both the 2021/2022 and 2022/2023 seasons. This unexpected wave of hospitalizations forced hospitals to reorganize departments and allocate personnel to meet the increased demand [29]. Although age and severity at admission were significantly higher in the post-COVID period, this did not translate into longer hospitalizations or increased use of invasive or non-invasive respiratory support.

Recent studies have described a higher incidence of bronchiolitis in the post‐pandemic period, but findings on disease severity are conflicting. Some studies align with our results [13, 30], while others, such as Brisca et al. [31], reported a substantial increase in the need for mechanical ventilation in previously healthy infants admitted for bronchiolitis during the 2022–2023 season. This discrepancy might be due to the setting of the studies; we included seven small/large PICUs, whereas Brisca’s single-center study was conducted in a large PICU that treats intubated patients from all over the region.

Our study confirmed that RSV infection is a significant risk factor for intubation, as reported by other authors [32], while coinfection and bacterial infection were not associated with the need for IMV. The number of patients with comorbidities has also significantly increased, reflecting a trend that has been observed for years in PICU admissions [33]. In our sample, one-fifth of the patients had at least one comorbidity, with the respiratory system being the most affected. This result could be underestimated, as bronchiolitis may unmask underlying cardiac, pulmonary, or neurological conditions, even in children previously considered healthy [34].

An interesting finding from our study is that the COVID-19 pandemic appeared to influence the risk of intubation in the PICUs included. Although the percentage of intubated children remained stable across all study years, multivariable analysis revealed a strong effect of the time period on intubation. Specifically, the pre-pandemic period acted as a protective factor, even after adjusting for age and disease severity at admission. Additionally, there is a “Center effect” of about 22% on the variability of the observed outcome, indicating that some PICUs intubate more frequently in the post-COVID era, despite similar clinical conditions. The reasons for this tendency are difficult to ascertain. As hospitals were reorganized to manage the COVID-19 pandemic, changes in staffing, such as lower nurses-to-patient ratios or other organizational modifications could have contributed [35, 36]. A multicenter Italian study reported a dramatic increase in the use of HFNC in pediatric wards during the first post-COVID season [37], which may have delayed PICU admissions. We attempted to understand the causes of this “Center effect” by categorizing PICUs into large and small centers, hypothesizing that centers of similar size might have similar organizational and staffing challenges, but no association between center size and the risk of intubation has been found.

Significant differences between centers were observed in ventilation practices, regardless of the pandemic. We observed a tenfold variation in the prevalence of intubation rate between PICUs. Schlapbach et al. reported similar results [38], noting that intubation rates were neither explained by the case mix nor by time trends and likely reflected underlying differences in unit-to-unit practice. These results highlighted a gap between scientific evidence and care practice, despite the continuous publication of guidelines on bronchiolitis management [39]. Factors such as nurse availability and training, already described as crucial for successful NIV management, may play a role in these differences [40].

Another interesting aspect, probably unrelated to the pandemic, is the change in interface and NIV mode usage over time. We observed a shift from nasal prongs to nasal masks as the preferred interface for NIV. A 2015 study conducted in Italy [41] on the use of NIV in acute respiratory failure (ARF) showed that most children—around 50%—received NIV through nasal prongs. Although the two scenarios are not directly comparable—we are considering only bronchiolitis cases, while Wolfler et al. [41] referred to any kind of ARF—our data suggest a change in interface preference over the years. The significant age difference between the two groups could justify the use of nasal masks in older patients. However, the available sample does not allow us to further analyze this issue. Another possible explanation is that concerns have been raised about asynchrony with nasal prongs [42] and associations between asynchrony and worse clinical outcomes have been described [43].

CPAP usage decreased from 44 to 31% between the two study periods, while PSV/BiPAP usage doubled. Compared to the 2015 study [41], where CPAP was the most commonly used NIV strategy in infants, in our cohort PSV/BiPAP represents 55% of total non-invasive strategies in the post-COVID period. This pattern is consistent with a European survey on NIV practices in children [44], which showed that PSV was the most commonly used bilevel ventilation mode, as observed in our cohort. Interestingly, only a few studies have evaluated the performance of BiPAP in bronchiolitis management [45].

This study has several limitations. It is based on a database analysis from a prospectively compiled national registry and missing data from available records may have influenced our analyses, although this bias has been controlled for by the study design. Furthermore, not all Italian PICUs could participate in the study, therefore, the potentially partial sample may limit the generalizability of the results obtained. Moreover, we did not collect the timing for intubation (e.g., before arriving in PICU) but we only knew whether the patient was intubated and the length of IMV. Finally, we have no data on blood gas analysis (apart from data present in the PIM-3 score), blood chemistry, or chest X-ray results because they were not collected in the TIPNet database.

Despite these limitations, we can conclude that our hospitals had to cope with an unexpected peak of significantly more severe cases of bronchiolitis, which, however, did not require greater respiratory support. We can speculate that the higher risk of intubation in the post-COVID period could be due to factors not only related to the clinical conditions of the patient but also to the specific center admitting the patient, with associated organizational, management, and staffing challenges. In light of these results, it seems necessary to strengthen the network at a national level to standardize the levels of care in each PICU and to outline uniform intubation criteria in cases of severe bronchiolitis.

## Supplementary Information

Supplementary Material 1: Supplementary Table S1. Clinical characteristics of admitted patients by center (2020/21 season included, N = 715). Underlined: lowest and highest number for every characteristic.

Supplementary Material 2: Supplementary Table S2. Univariable logistic regression models for intubation outcome.

## Data Availability

No datasets were generated or analysed during the current study.
