# Supplementary material for: Severe bronchiolitis before and after the COVID-19 pandemic: a retrospective database analysis by the Italian Network of PICU study group (TIPNet)
Source: J Anesth Analg Crit Care. 2024 Nov 26;4:78. doi: 10.1186/s44158-024-00210-1 (PMC11600936; doi:10.1186/s44158-024-00210-1)
Supplement: Supplementary file 1 — Supplementary Material 1: Supplementary Table S1. Clinical characteristics of admitted patients by center (2020/21 season included, N = 715). Underlined: lowest and highest number for every characteristic. [file 44158_2024_210_MOESM1_ESM.docx]

| Characteristic | N | 1  N = 85 | 2  N = 53 | 3  N = 39 | 4  N = 138 | 5  N = 73 | 6  N = 83 | 7  N = 244 | Overall,  N = 715 |
| --- | --- | --- | --- | --- | --- | --- | --- | --- | --- |
| Gender (M) | 715 | 55 (65%) | 33 (62%) | 18 (46%) | 76 (55%) | 39 (53%) | 41 (49%) | 129 (53%) | 391 (55%) |
| Age (d), median(IQR) | 715 | **50 (34, 104)** | **125 (35, 347)** | 101 (62, 168) | 74 (46, 210) | 60 (36, 151) | 53 (22, 136) | 53 (28, 111) | 64 (34, 146) |
| PIM 3 score (%), median(IQR) | 686 | 0.45 (0.20, 0.56) | 0.47 (0.37, 0.65) | **0.16 (0.12, 0.51)** | 0.46 (0.41, 0.54) | 0.43 (0.19, 0.76) | 0.46 (0.27, 0.63) | **0.53 (0.45, 0.68)** | 0.48 (0.37, 0.63) |
| Comorbidities | 712 | **4 (4.7%)** | 12 (23%) | 4 (10%) | 25 (18%) | 16 (22%) | **22 (27%)** | 35 (15%) | 118 (17%) |
| Ex-premature | 715 | **5 (5.9%)** | 6 (11%) | 5 (13%) | 8 (5.8%) | 8 (11%) | **15 (18%)** | 36 (15%) | 83 (12%) |
| NIV | 620 | 52 (91%) | **23 (50%)** | 26 (74%) | **122 (95%)** | 32 (64%) | 48 (66%) | 197 (85%) | 500 (81%) |
| IMV | 620 | 5 (8.8%) | **23 (50%)** | 9 (26%) | **6 (4.7%)** | 18 (36%) | 25 (34%) | 34 (15%) | 120 (19%) |
| PSV/BiPAP | 475 | 14 (35%) | 8 (29%) | **1 (3.4%)** | 51 (42%) | 1 (2.6%) | **38 (78%)** | 64 (38%) | 177 (37%) |
| CPAP | 475 | 26 (65%) | 20 (71%) | **28 (97%)** | 57 (47%) | 32 (82%) | 7 (14%) | **15 (8.9%)** | 185 (39%) |
| A-PCV | 475 | **0 (0%)** | **0 (0%)** | **0 (0%)** | 13 (11%) | 4 (10%) | 4 (8.2%) | **90 (53%)** | 111 (23%) |
| NIV length (d), median(IQR) | 576 | 3.00 (2.00, 5.00) | 2.00 (2.00, 3.00) | 3.00 (2.00, 4.00) | 3.00 (2.00, 4.00) | 3.00 (2.00, 4.00) | 2.00 (1.00, 4.00) | 3.00 (2.00, 6.00) | 3.00 (2.00, 5.00) |
| IMV length (d), median(IQR) | 119 | 11 (9, 13) | 8 (5, 12) | 7 (4, 13) | 4 (3, 5) | 11 (9, 14) | 7 (5, 11) | 12 (8, 16) | 10 (6, 14) |
| PICU LOS (d), median(IQR) | 715 | 5.0 (4.0, 6.0) | 5.0 (3.0, 8.0) | 5.0 (4.0, 6.5) | 4.0 (3.0, 5.0) | 4.0 (3.0, 7.0) | 4.0 (2.0, 8.0) | 5.0 (3.0, 9.0) | 4.0 (3.0, 7.0) |

Table I supplementary: Clinical characteristics of admitted patients by center (2020/21 season included, N= 715). **Underlined**: lowest and highest number for every characteristic.

Abbreviations: PIM, Pediatric Index of Mortality; PICU, Pediatric Intensive Care Unit; NIV, Non-Invasive Ventilation; IMV, Invasive Mechanical Ventilation; PSV, Pressure Support Ventilation; BiPAP, Bilevel Positive Airway Pressure; CPAP, Continuous Positive Airway Pressure; A-PCV, Assisted Pressure Controlled Ventilation; LOS, Length of Stay.
