# Supplementary material for: Severe bronchiolitis before and after the COVID-19 pandemic: a retrospective database analysis by the Italian Network of PICU study group (TIPNet)
Source: J Anesth Analg Crit Care. 2024 Nov 26;4:78. doi: 10.1186/s44158-024-00210-1 (PMC11600936; doi:10.1186/s44158-024-00210-1)
Supplement: Supplementary file 2 — Supplementary Material 2: Supplementary Table S2. Univariable logistic regression models for intubation outcome. [file 44158_2024_210_MOESM2_ESM.docx]

Table II Supplementary. Univariable logistic regression models for intubation outcome.

| *Predictors* | *Odds Ratios* | *CI* | *p* |
| --- | --- | --- | --- |
| Pre-COVID period | 0.90 | 0.58 – 1.37 | 0.616 |
| Age (months) | 0.96 | 0.92 – 1.00 | **0.042** |
| Large center^ | 0.56 | 0.37 – 0.86 | **0.008** |
| Season^§^ |  |  |  |
| Season 2018-1019 | 1.46 | 0.79 – 2.73 | 0.226 |
| Season 2019-1020 | 1.08 | 0.59 – 1.98 | 0.799 |
| Season 2021-1022 | 1.21 | 0.64 – 2.30 | 0.561 |
| Season 2022-1023 | 1.45 | 0.73 – 2.93 | 0.295 |
| PIM3 score | 0.92 | 0.80 – 0.99 | 0.128 |
| Comorbidity | 0.61 | 0.37 – 1.02 | 0.054 |
| Ex-prematurity | 0.57 | 0.33 – 1.00 | **0.043** |
| A-PCV/BiPAP/PSV | 0.82 | 0.41 – 1.59 | 0.567 |
| Nasal mask | 6.73 | 3.28 – 16.24 | **<0.001** |
| Facial Mask | 0.30 | 0.12 – 0.74 | **0.007** |
| Nasal prongs | 5.51 | 3.43 – 9.19 | **<0.001** |
| Helmet | 2.70 | 1.29 – 6.60 | **0.015** |
| Full face | 0.69 | 0.34 – 1.52 | 0.321 |
| RSV infection | 2.25 | 1.34 – 3.72 | **0.002** |
| Co-infection | 0.31 | 0.19 – 0.52 | **<0.001** |
| Bacterial superinfection | 0.09 | 0.05 – 0.17 | **<0.001** |

^^^ Reference: PICUs with less than 6 beds

^§^ Reference: Season 2017-2018Abbreviations: CI, Confidence Interval; PIM, Pediatric Index of Mortality; PICU, Pediatric Intensive Care Unit; PSV, Pressure Support Ventilation; BiPAP, Bilevel Positive Airway Pressure; A-PCV, Assisted Pressure Controlled Ventilation; RSV, Respiratory Syncytial Virus
